# Molecular characterization and functional analysis of barley semi-dwarf mutant Riso no. 9265

**DOI:** 10.1186/s12864-015-2116-x

**Published:** 2015-11-14

**Authors:** Qiaojun Jia, Chengdao Li, Yi Shang, Jinghuan Zhu, Wei Hua, Junmei Wang, Jianming Yang, Guoping Zhang

**Affiliations:** Institute of Crop Science, Zhejiang University, Hangzhou, 310058 China; Institute of Crop and Nuclear Technology Utilization, Zhejiang Academy of Agricultural Sciences, Hangzhou, 310021 China; Western Barley Genetics Alliance, Murdoch University, Murdoch, WA6150 Australia

**Keywords:** Barley, Deletion, Gene compensation, Gibberellin, Semi-dwarf

## Abstract

**Background:**

*sdw1/denso* is one of the most important and useful semi-dwarf genes in barley breeding. At least four *sdw1/denso* alleles have been reported and *HvGA20ox*_*2*_ is considered as the candidate gene. Up to date, results of studies have not univocally proven the genetic relationship between *sdw1/denso* and *HvGA20ox*_*2*_*.*

**Results:**

In the present study, a complete deletion of Morex_contig_40861 including both *HvGA20ox*_*2*_ and Mloc_56463 genes was identified at the *sdw1* locus from a semi-dwarf mutant Riso no. 9265. Expression of the genes encoding gibberellin biosynthesis (*HvGA20ox*_*1*_ and *HvGA3ox*_*2*_) were increased in the mutant compared to the wild type Bomi, while the expression of GA catabolic gene *HvGA2ox*_*3*_ was decreased. Over-expression of *HvGA20ox*_*2*_ could rescue the semi-dwarf phenotype and increase GAs concentration.

**Conclusions:**

We confirmed that a GA biosynthetic enzyme HvGA20ox_2_, acted as GA 20-oxidase, is the functional gene for the *sdw1/denso* semi-dwarfism. Lose of *HvGA20ox*_*2*_ is partially compensated by *HvGA20ox*_*1*_ and further feedback is regulated by gibberellin. We also deduced that the *sdw1/denso* allele itself affects later heading owing to its reduced endogenous GAs concentration.

**Electronic supplementary material:**

The online version of this article (doi:10.1186/s12864-015-2116-x) contains supplementary material, which is available to authorized users.

## Background

Plant height is one of the most important agronomic traits in cereal crops that not only determines plant architecture, but also is closely associated with grain yield. A reduction in plant height usually leads to strong straw and high resistance to lodging, as happened in semi-dwarf rice and wheat cultivars. However, reduced plant height means lowering of canopy, which favors the epidemic spread of fungal diseases, resulting in an undesired increase of fungicide use, and a reduction of yield potential mainly due to smaller grain weight [1]. For optimizing the balance of plant height and yield, breeders have successfully utilized the semi-dwarf genes in cereal crop breeding since 1960s. The development and wide cultivation of semi-dwarf wheat and rice varieties led to a dramatic increase of cereal production worldwide [2], which is labeled as a ‘green revolution’.

The Green Revolution genes have been identified and isolated in the semi-dwarf varieties by reversed genetics. Among them, *sd1* in rice is one of the most famous genes and has been widely used to produce semi-dwarf phenotype in both *japonica* and *indica* rice. It was reported that *sd1* encoded one of the GA 20-oxidases and was involved in the last steps of gibberellin biosynthesis [3–6]. Semi-dwarf genes have also been extensively explored in barley breeding programs and more than 30 types of them have been described [7]. One of the most successfully used semi-dwarf genes in modern barley breeding is *sdw1/denso*, which was postulated as homologous to *ga5* in *Arabidopsis* and *sd1* in rice [8, 9]. The main phenotypic effect of the *sdw1/denso* gene is a 10-20 cm reduction of plant height, depending on environmental conditions [10]. Besides, there are some deleterious effects associated with *sdw1/denso* gene, such as late heading and maturity, decreased thousand-grain weight and high screening [10–15]. As for the relationship between grain yield and *sdw1/denso*, both positive and negative were reported depending on different genetic backgrounds [10, 11, 15]. However, with its suitable semi-dwarf phenotype and potentially increased yield, *sdw1/denso* has been introduced to numerous cultivars. For example, newly released more than 150 European cultivars carried the *denso* allele [16].

There are at least four independent alleles based on the allelism test done up to date [12, 17]. One spontaneous mutant was derived from a Danish variety Abed *denso* in 1946, as named accordingly [17]. Two alleles induced by X-ray with different parents were named as *denso* and *sdw1*, respectively. Interestingly, the *denso* mutant has been used to develop malting barley, while the *sdw1* has been limited to feed barley [10, 12]. The fourth *sdw1* allele was found in a M_2_ generation from the variety Bomi, treated by neutrons in the Stockholm reactor and named as Riso no. 9265. Although these mutants are characterized by semi-dwarf phenotype, all of them are sensitive to GA_3_ [18].

In 1993, *sdw1/denso* was first mapped on the 3HL as a phenotypic trait [19]. In our previous study, *GA20ox*_*2*_ homolog was identified using PCR method with the primers designed from the conserved domain of rice *sd1* and considered as a candidate gene of *sdw1/denso* [8]. Meanwhile, it was found that the expression of *HvGA20ox*_*2*_ in *denso* mutant was reduced by 4-fold, but almost 60-fold in the *sdw1* mutant, compared to the control [15]. However, there was no direct evidence to prove the *HvGA20ox*_*2*_ as the functional gene. In addition, the function of *HvGA20ox*_*2*_ was predicted based on its functional domain, being highly similar with those of *sd1* (*OsGA20ox*_*2*_) and *ga5* (*AtGA20ox*_*1*_). No evidence showed its function *in vitro* or *in vivo*. In the present work we investigated the *sdw1* allele of Riso no. 9265 and identified a complete deletion contig, which includes two genes Mloc_56462 (*HvGA20ox*_*2*_) and Mloc_56463 (a putative methyltransferase PMT26). The function of the *HvGA20ox*_*2*_ gene was further analyzed using genetically-transformed *Arabidopsis*. Furthermore, real-time PCR assays revealed that transcript level of GA synthesis-related genes were significantly different between Bomi and Riso no. 9265. The current results showed that the semi-dwarf phenotype of Riso no. 9265 is attributed to the deletion of *HvGA20ox*_*2*_.

## Results

### Plant height, internode length and heading date of Bomi and Riso no. 9265

At maturity, plant height and length of spike, culm and internodes of Bomi and its semi-dwarf mutant Riso no. 9265 were measured (Table 1). Plant height of Bomi was 83.1 cm, and Riso no. 9265 was 63.3 cm, being only 76.2 % of its wild type parent, as reported previously [17]. No obvious difference was found in spike length between the two genotypes. Obviously, the reduction of plant height for the mutant is completely attributed to the shorter culm length. Culm length of Riso no. 9265 was 53.5 cm, being 19.6 cm shorter than that of Bomi. In fact, all internodes of the mutant were shorter than those of the wild type (Table 1). Moreover, the difference between the two genotypes was smaller for the length of the top two internodes and larger for the basal four internodes. Therefore, it may be assumed that the effect of the semi-dwarf gene in Riso no. 9265 is mainly on the basal internodes. In addition, Riso no. 9265 headed three days later than Bomi.

**Table 1:** Internode length and plant height of Bomi and Riso no. 9265

Traits	Bomi	%	Riso no.9265	%
	Length (cm)		Length (cm)	
Plant height	83.2 ± 1.1		63.5 ± 2.4**	
Spike length	10.1 ± 0.4		10.0 ± 0.5	
Culm height	73.1 ± 0.9	100	53.5 ± 2.1**	100
First-internode length	25.2 ± 1.0	34.5	20.1 ± 0.7*	37.6
Second-internode length	14.9 ± 0.3	20.4	13.1 ± 0.7*	24.5
Third-internode length	10.5 ± 0.4	14.4	7.6 ± 0.6**	14.2
Fourth-internode length	10.8 ± 0.4	14.8	6.7 ± 0.4**	12.5
Fifth-internode length	8.2 ± 0.6	11.2	4.2 ± 0.3**	7.9
Sixth-internode length	3.5 ± 0.6	4.8	1.8 ± 0.2**	3.4

Values are means ± SE, N = 20. *significant difference at 0.005 level; **significant difference at 0.001 level

### *HvGA20ox*_*2*_ was deleted in Riso no. 9265

**Fig. 1:**
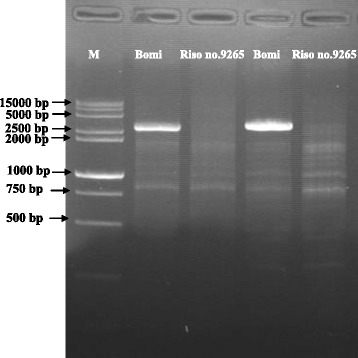
Dectection of the *HvGA20ox*_*2*_ gene by PCR analysis. M, Marker; the right two lanes were produced by forward primer sdwF10 and reverse primer sdwR10; the left two lanes were produced by forward primer sdw9 and reverse primer sdwR13

In this study, the complete genome sequence of *HvGA20ox*_*2*_ (Mloc_56462) was identified after blast against Gramene (http://www.gramene.org/) using the sequences of *HvGA20ox*_*2*_ from our previous study [8]. Two pairs of primers of *HvGA20ox*_*2*_ were designed to amplify its genomic sequence. In Bomi, both primer pairs produced single band with different sizes. One is 2974 bp and the other is 3165 bp. However, no PCR product was obtained in Riso no. 9265 (Fig. 1), indicating that *HvGA20ox*_*2*_ is lost in the mutant. Thus, the sequences of *HvGA20ox*_*2*_ were used to identify Contigs after blast against International Barley Genome Sequencing Consortium (http://webblast.ipk-gatersleben.de/barley/viroblast.php). One Morex_contig_40861, covering 21596 bp was identified, and it contains *HvGA20ox*_*2*_ and Mloc_56463 genes. Several primers covering Morex_contig_40861 were designed (Additional file 1: Table S1) and PCR amplification succeeded in Bomi, but failed in the mutant Riso no. 9265. Furthermore, we were able to amplify flanking genes (Mloc_3247, Mloc_45089, Mloc_51746 and Mloc_7311) of Morex_contig_40861, obtained using the latest barley assembly from Gramene and barley reference genome information from Leibniz Institute of Plant Genetics and Crop Plant Research (IPK) (primers are listed in Additional file 1: Table S1). As a result, it was found that there is a large deletion spanned at least two genes *HvGA20ox*_*2*_ and Mloc_56463 in Riso no. 9265. A blast search for the Mloc_56463 protein sequence against NCBI identified a possible methyltransferase PMT26-like gene.

### Overexpression of *HvGA20ox*_*2*_ caused more gibberellin production in *Arabidopsis*

**Fig. 2:**
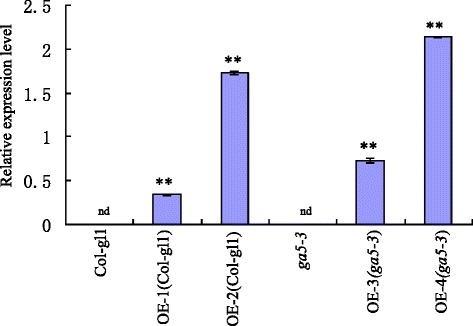
Relative expression level of *HvGA20ox*_*2*_ in transgenic *Arabidopsis.* nd, not detected. **means significant difference at 0.01 level. nd means not detected

To evaluate if *HvGA20ox*_*2*_ is responsible for the semi-dwarf phenotype, we examined transgenic *Arabidopsis* plants that over-express *HvGA20ox*_*2*_ in wild type (Col-gl1) and a semi-dwarf mutant *ga5-3*. T1 transgenic plants were selected by BASTA analysis and PCR method. We obtained eleven and six transgenic lines in Col-gl1 and *ga5-3* background, respectively. All of them displayed a higher growth rate. Homozygous T3 plants of transgenic Col-gl1 lines (OE-1 and OE-2) and transgenic *ga5-3* lines (OE-3 and OE-4) were taken randomly for further analysis. The four transgenic plants differed greatly in the expression levels of *HvGA20ox*_*2*_. However, transcripts of barley GA oxidase-encoding gene were not found in both wild type and semi-dwarf mutant *ga5-3* (Fig. 2).

**Fig. 3:**
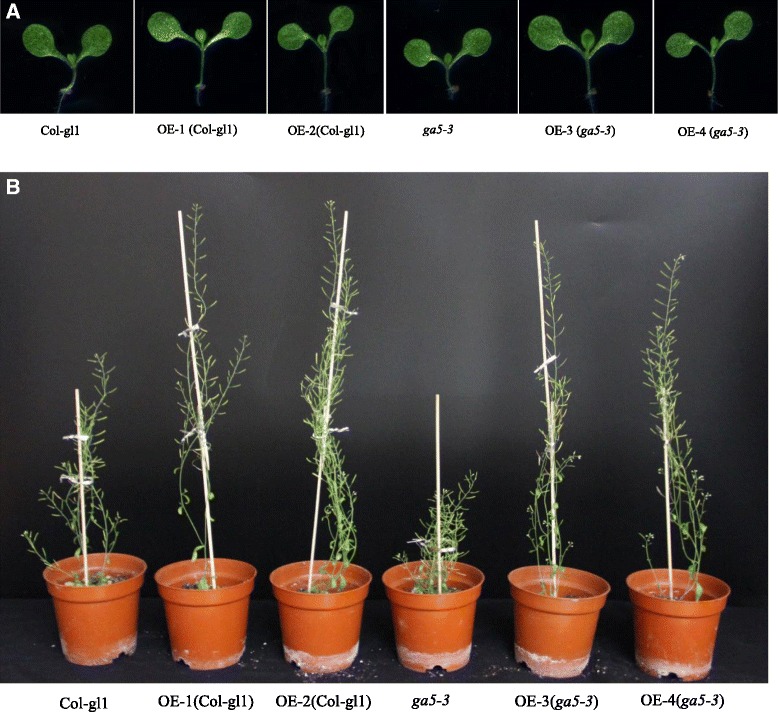
Overexpression of *HvGA20ox*_*2*_ in *Arabidopsis* affects plants development. The pictures compared the growth of transgenic and untransgenic at 1-week (**a**) and 6-week (**b**)

The two independent transgenic lines (OE-1 and OE-2) showed GA-overdose phenotype although they differed in the expression level of *HvGA20ox*_*2*_. There was no obvious difference in root length of 7-day-old seedlings between wild type and *HvGA20ox*_*2*_ over-expression plants, but the latter had 50 % longer hypocotyl than the former (Table 2 and Fig. 3). In addition, the transgenic plants flowered relatively early.

**Table 2:** Phenotypic parameters for wild type, *ga5-3* and *HvGA20ox*_*2*_ over-expressing transgenic *Arabidopsis*

Traits	Col-gl1	OE-1(Col-gl1)	OE-2(Col-gl1)	*ga5-3*	OE-3(*ga5-3*)	OE-4(*ga5-3*)
Hypocotyl length (mm)	2.05 ± 0.04	3.64 ± 0.10A	4.04 ± 0.01A	2.07 ± 0.04A	3.64 ± 0.10AB	3.75 ± 0.12AB
Root length (mm)	36.1 ± 0.7	38.9 ± 1.8	40.1 ± 1.2	37.5 ± 0.67	40.3 ± 1.4	36.8 ± 1.7
Flowering time (d)	26.2 ± 0.35	24.6 ± 0.33A	23.0 ± 0.25A	26.7 ± 0.28	22.5 ± 0.23AB	23.7 ± 0.24AB
Vegetative internode length (cm)	1.9 ± 0.1	2.7 ± 0.1A	2.9 ± 0.1A	0.6 ± 0.1A	3.0 ± 0.2AB	2.7 ± 0.1AB
No.vegetative internodes	3.4 ± 0.1	5.2 ± 0.2A	5.0 ± 0.2A	3.1 ± 0.1A	4.8 ± 0.4AB	4.9 ± 0.1AB
inflorescence internode length (cm)	0.61 ± 0.01	0.68 ± 0.01A	0.71 ± 0.02A	0.38 ± 0.01A	0.67 ± 0.02AB	0.64 ± 0.02AB
No.inflorescence internodes	34.4 ± 1.1	47.3 ± 1.2A	43.9 ± 1.4A	31.5 ± 1.1A	43.7 ± 1.6AB	47.3 ± 1.9AB
Final plant (cm)	28.8 ± 0.5	46.8 ± 0.7A	44.8 ± 1.0A	14.6 ± 0.4A	42.0 ± 1.7AB	43.8 ± 1.1AB

The values are the means ± SE

Capital letter A means significantly different from the wild type (Col-gl1); Capital letter B means significantly different from *ga5-3* mutant

**Fig. 4:**
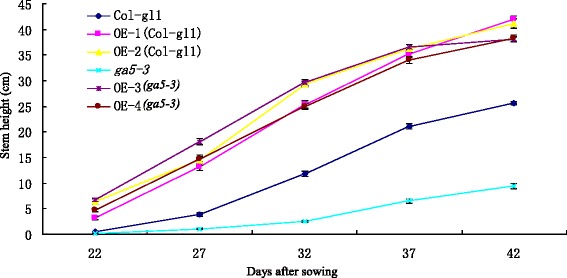
Stem heights of *Arabidopsis* over expressing *HvGA20ox*_*2*_ gene

To determine whether the over-expression constructs would affect the phonotype of *ga5-3*, we characterized the GA-affecting traits in *ga5-3* transgenic lines (OE-3 and OE-4), including hypocotyl length, internodes length and number, flowering time, and final plant height. The results showed that *ga5-3* transgenic lines (OE-3 and OE-4) also had the GA-overdose phenotype, with longer hypocotyls and internodes, more internode number, flowering earlier in comparison with those of wild type or *ga5-3* mutant (Table 2 and Fig. 3). It can be seen from Fig. 4 that there was the dramatic difference in plant height among the four transgenic lines, wild type and *ga5-3* throughout the whole growth stage.

**Fig. 5:**
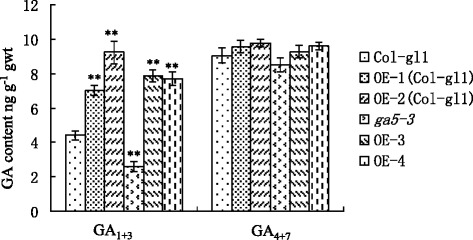
The content of active gibberellin in the 7-d-old seedlings of transgenic and control plants. Each column represents the mean of three repeats with ±SE bar. **means significant difference at 0.01 level

GA_4+7_ content were much higher than GA_1+3_ content in wild type. The transgenic plants had significantly higher GA_1+3_ content than both wild-type and *ga5-3* plants, but the difference in GA_4+7_ content among them was much smaller. Moreover, GA_1+3_ content was lower in *ga5-3* mutants than in wild-type plants, whereas both genotypes had the similar GA_4+7_ content (Fig. 5).

**Fig. 6:**
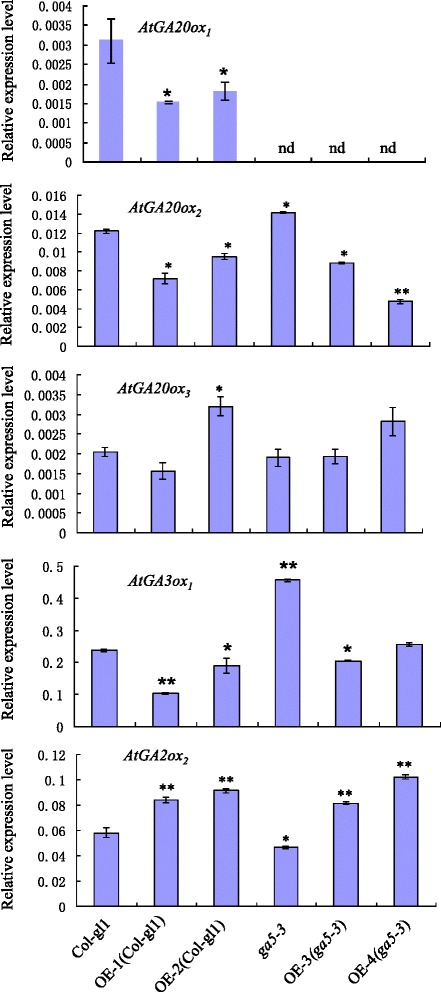
The effect of over expression *HvGA20ox*_*2*_ on expression of gibberellin biosythetic and catabolic genes. *indicades significant difference at 0.05 level; **means significant difference at 0.01 level.; nd means not detected

In order to determine whether the changes of phenotype and GA content observed in the transgenic lines are accompanied by the alteration in expression of GA biosynthesis and catabolism, three GA 20-oxidases, one GA 3-oxidase and one GA 2-oxidase, highly expressed in 7-day-old seedlings, were selected for further analysis. As shown in Fig. 6, the expression of *AtGA20ox*_*2*_ and *AtGA3ox*_*1*_ was greatly up-regulated in the *AtGA20ox*_*1*_ deficient mutant *ga5-3*, while the expression of *AtGA2ox*_*2*_ was significantly decreased in comparison with the wild type. No significant difference was found in the expression of *AtGA20ox*_*3*_ between *ga5-3* and wild type. In the transgenic *Arabidopsis* with *HvGA20ox*_*2*_ over-expression, the expression of *AtGA20ox*_*1*_, *AtGA20ox*_*2*_ and *AtGA3ox*_*1*_ was distinctly decreased, however, *AtGA2ox*_*2*_ was dramatically increased relative to wild type and *ga5-3* mutants.

### The expression of GA biosynthesis and catabolism genes were changed in Riso no. 9265 mutant

**Fig. 7:**
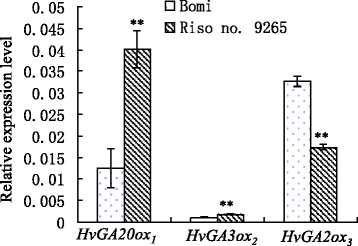
Transcriptional changes of barley GA biosynthetic and catabolic genes in stem at the initial jointing stage. **means significant difference at 0.01 level

We determined the expression level of GA dioxygenase genes in stems of Bomi and Riso no. 9265 at the initial jointing stage. *HvGA20ox*_*1*_, *HvGA3ox*_*2*_ and *HvGA2ox*_*3*_ showed high transcript level. The relative mRNA expression of *HvGA20ox*_*1*_ and *HvGA3ox*_*2*_ were dramatically increased in the mutant Riso no. 9265 compared with those of Bomi. On the other hand, the mRNA expression of *HvGA2ox*_*3*_ was decreased in the Riso no. 9265 (Fig. 7).

## Discussion

Previously, we reported that barley *HvGA20ox*_*2*_ (Mloc_56462) was a candidate gene of *sdw1/denso* and different expression level of *HvGA20ox*_*2*_ was observed between *denso* and *sdw1* (Jotun) alleles [8, 15]. Here, we reported that around 21 kb DNA fragment, including both *HvGA20ox*_*2*_ and Mloc_56463 genes was completely deleted in the mutant Riso no. 9265. Because of the incomplete barley reference genomes and repetitive nature of the barley genome [20], the boundary of deleted segments was not determined precisely in Riso no. 9265. Gene annotation showed that *HvGA20ox*_*2*_ has gibberellin 20-oxidase activity and may be involved in GA biosynthetic pathway. Moreover, the *sdw1/denso* mutant is GA sensitive and exogenous application of 10 ppm GA_3_ restored its plant height [18], which indicates that *sdw1/denso* mutants are GA deficiency. On the contrary, Mloc_56463 is a putative methyltransferase and has not been reported or predicted as a GA-related gene. Thus, *HvGA20ox*_*2*_ is considered as the only candidate responsible for the semi-dwarfism in Riso no. 9265.

The GA biosynthetic pathway has been extensively investigated and the enzymes involved have been well characterized in plants [21]. The early GA-biosynthetic steps are encoded by single-copy genes, while the final steps catalyzed by GA 20-oxidase, GA 3-oxidase and GA 2-oxidase, are encoded by multigene families. GA20ox activity removes carbon-20 in the formation of C19-GA skeleton [22, 23]. In *Arabidopsis*, GA20-oxidase genes are differentially expressed and involved in different developmental processes controlled by GA, but the plants that constitutively express each of three *GA20oxs* (*AtGA20ox*_*1*_, *AtGA20ox*_*2*_ and *AtGA20ox*_*3*_) behave as the control treated with GA [22, 23]. The GA-over-production phenotype was also found in *HvGA20ox*_*2*_ transgenic *Arabidopsis* (Table 2, Figs. 3 and 4). The phenotype was characterized by elongated hypocotyl and stems, early flowering, and higher growth rate. In addition, barley *GA20ox*_*2*_ could recover *AtGA20ox*_*1*_ lose-of-function mutant. These results suggest that *HvGA20ox*_*2*_ is the orthologous of *AtGA20ox*_*1*_ and acts as the oxidase at carbon-20 of GA biosynthetic pathway.

The bioactive GAs in plants are GA_1_, GA_3_, GA_4_ and GA_7_, but GA_4_ is a major active GA in *Arabidopsis* [24]. Both endogenous GA_1+3_ and GA_4+7_ in 7-day-old seedlings of the control and transgenic plants were quantified in this study. The results showed that transgenic plants had higher GA contents, especially for GA_1+3_ compared with the control. The increased GAs is associated with the changed phenotype (Figs. 3 and 4). Similar results have been reported that GA_1_ content was increased and GA_4_ content had little change in the 7-day-old *Arabidopsis* with *AtGA20ox*_*1*_ overexpressors [22]. In contrast, both GA_1_ and GA_4_ content had slight change in the shoot tip of *Arabidopsis* plant with over-expression *AtGA20ox*, while a 2- to 3-fold increase in GA_4_ content was observed in the rosette leaves of the transgenic lines [23]. Radi et al. [25] deduced that the dramatic difference in GAs content might be due to the variation in GA level among plant tissues and growth stage.

As indicated in Fig. 6, the transgenic lines overexpressing barley GA 20-oxidases showed a decrease in *AtGA20ox*_*1*_, *AtGA20ox*_*2*_, *AtGA3ox*_*1*_ and an increase in *AtGA2ox*_*2*_ expression, while *ga5-3* was just opposite in the expression of these genes, which could be attributed to feedback from an increased bioactive GAs in *HvGA20ox*_*2*_ over-expressing transgenic *Arabidopsis* or decreased bioactive GAs in *ga5-3*, respectively. A similar regulatory role has been proposed for *AtGA3ox*_*1*_, whose transcript level is also feedback-regulated in transgenic *Arabidopsis* of over-expressed *GA20ox* [23, 26]. No considerable change was detected in *AtGA20ox*_*3*_ level of transgenic *Arabidopsis* and the control, suggesting that *AtGA20ox*_*3*_ gene might be less sensitive than *AtGA20ox*_*1*_ and *AtGA20ox*_*2*_ to the alteration of bioactive GAs.

However, not all over-expressing *GA20ox* plants displayed GA-overdose phenotype. In the case of *CmGA20ox1*, over-expression of *Cucurbita maxima* GA 20-oxidase in *Arabidopsis* resulted in accumulation of inactive GA_25_ and GA_17_, and reduction of GA_4_ content, which caused a slight decrease in stem elongation [25, 27]. Accordingly, GAs accumulation and changed expression levels of GA-regulated transcripts confirmed that *HvGA20ox*_*2*_ should be involved in regulation of GAs production. In barley semi-dwarf mutant Riso no. 9265, lose of *HvGA20ox*_*2*_ caused its GA deficiency and reduced plant height.

It was reported that GA promoted stem elongation and was found in the young tissues [22, 23]. *HvGA20ox*_*2*_ is highly expressed in stem and possiblely has effect on internode length [15]. Once jointing stage starts, the stems are young and internode region begins to elongate and grow rapidly. Therefore, the transcriptional levels of GA biosynthetic and catabolic genes were detected at the initial jointing stage. As a result, we found that GA biosynthesis genes (*HvGA20ox*_*1*_ and *HvGA3ox*_*2*_) were up-regulated and GA catabolic gene (*HvGA2ox*_*3*_) was down-regulated in Riso no. 9265 compared with Bomi, suggesting a type of feedback regulation from the low bioactive GAs because of the null mutation of *HvGA20ox*_*2*_. Feedback regulation of *GA20ox* and *GA3ox* and feed-forward regulation of *GA2ox* genes expression have been also shown in GA-deficient *Arabidopsis* mutants [27–29]. In rice *sd1*, the expression of *OsGA20ox*_*1*_ was increased in stem to compensate the *sd1* effect [5, 30]. As a result, the increased expression of *HvGA20ox*_*1*_ could compensate the effect of *HvGA20ox*_*2*_ in the stem at least partially, and the feedback or feed-forward regulation of the GA dioxygenase helps maintaining a relative stable endogenous GA level. Consequently, Riso no. 9265 exhibited the observed plant height.

As mentioned above, we have characterized the barley semi-dwarf gene, *sdw1/denso*, and conclude that it encodes a GA biosynthetic enzyme, GA20ox, on the basis of the following results. Firstly, *sdw1/denso* mutant responds to exogenous GA_3_ [15, 18]. Secondly, the fourth *sdw1* allele is a complete lose of HvGA20ox_2_ activity due to the deletion of *HvGA20ox*_*2*_ gene in Riso no. 9265 (Fig. 1). In contrast, *denso* allele and *sdw1* of Jotun shows different expression of *HvGA20ox*_*2*_ [15]. Thirdly, the transgenic wild type *Arabidopsis* and semi-dwarf mutant *ga5-3* by *HvGA20ox*_*2*_ gene showed GA over-dose phenotypes. Finally, GA-regulated transcripts were changed in Riso no. 9265 in the same way as those of some GA20oxs in rice and *Arabidopsis* [5, 23, 26, 30].

It was reported that *sdw1/denso* was associated with later heading [11, 31]. Upon first mapped *denso* to the long arm of chromosome 3H, Barua et al. [19] found a quantitative trait locus for heading date, which could not be genetically separated from the *denso* locus. Previously identified QTLs of heading date was also located on the region around *denso* in the Blenheim × E224/3 DH population [14]. Furthermore, it was found that *sdw1* delayed maturity by around 3d based on eight populations [10]. We also found the QTL of development score was co-located with the *HvGA20ox*_*2*_ eQTL on 3HL [15]. However, it is still difficult to distinguish if it is pleiotropy of *sdw1/denso* or a tight linkage between the gene and one controlling later heading. In the present study, over-expression of *HvGA20ox*_*2*_ caused early flowering in *Arabidopsis*. The same is true for *AtGA20oxs* over-expressors [22, 23]. Both *AtGA20ox*_*1*_ and *AtGA20ox*_*2*_ act redundantly and affect many developmental processes, with *AtGA20ox*_*1*_ making the great contribution to internode and filament elongation, and *AtGA20ox*_*2*_ making the great contribution to flowering time and silique length in *Arabidopsis* [29]. It was also revealed that either *AtGA20ox*_*1*_ or *AtGA20ox*_*2*_ mutation delayed flowering under short-day condition,while only *AtGA20ox*_*2*_ delayed flowering slightly under long-day condition. Thus it can be assumed that the deficiency of *AtGA20ox*_*1*_ or *AtGA20ox*_*2*_ together with low GA affects flowering time, because GA acts a particularly important developmental switch between vegetative and reproductive development [32]. In the same way, it might be the deletion of *HvGA20ox*_*2*_ that causes later heading due to low GA concentration in Riso no. 9265. The hypothesis could be proved by the following facts. Firstly, GAs is involved in many developmental processes, including germination, stem extension, flowering and fruit set [22, 23, 29, 32]. Secondly, *HvGA20ox*_*2*_ and Mloc_56463 are absent in Riso no. 9265, unlike its wild type, while it is *HvGA20ox*_*2*_ that has gibberellin 20-oxidase activity and acts as a major determinant for GA production. Thirdly, GA deficiency was proved in *sdw1/denso* using GA sensitive experiment [15, 18]. Lastly, Riso no. 9265 mutant exhibited a late heading date. Similar results were found that semi-dwarf progenies with *sdw1/denso* matured generally later than their tall counterparts [10, 11, 31]. Considering all of these, it can be concluded that late heading in *sdw1/denso* could be the pleiotropy of *HvGA20ox*_*2*._

## Conclusions

The current study showed a complete deletion of over 21 kb DNA fragment including both *HvGA20ox*_*2*_ and Mloc_56463 genes in the mutant Riso no. 9265. *HvGA20ox*_*2*_ acts as GA 20-oxidase and is involved in gibberellin biosynthesis. The expression of the genes encoding GA biosynthesis (*HvGA20ox*_*1*_ and *HvGA3ox*_*2*_) are up-regulated and the expression of GA catabolic gene *HvGA2ox*_*3*_ is down-regulated in Riso no. 9265 in comparison with those in wild type Bomi, respectively. We conclude that *sdw1/denso* encodes one of the GA biosynthetic enzymes, GA 20-oxidase and the deletion of *HvGA20ox*_*2*_ as well as the compensatory of *HvGA20ox*_*1*_ and feedback regulation of gibberellin results in semi-dwarf phenotype of Riso no. 9265. We also deduced that *sdw1/denso* allele itself evoked later heading due to its reduced endogenous GAs concentration.

## Methods

### Plant materials and sampling

Semi-dwarf mutant Riso no. 9265, kindly provided by Dr. Chengdao Li of Murdoch University, Australia and its wild parent Bomi, kindly provided by Dr. Jing Zhang of Chinese Academy of Agricultural Sciences, China were used in this study. Both genotypes were planted in 2 × 6 m plots. The heading date was recorded as the number of days after sowed when 50 % of the spike emerged from the sheath. At maturity, 20 plants of each genotype were harvested at random for measurement of plant height, spike and internode length. Meanwhile, main stems of each genotype were taken at the initial jointing stage for measuring the expression levels of gibberellin biosynthetic and catabolic genes.

### DNA isolation, PCR amplification and sequencing

Seedlings at tillering stage were sampled and DNA was extracted according to Murray and Thompson [33] with small modification. DNA samples were quantified using a Thermo Unicam UV300 and adjusted to a final concentration of 50 ng/μl for PCR analysis. PCR was performed on a Veriti 96 well thermocycler (Applied Biosystems). Sequencing of *HvGA20ox*_*2*_ isolated from barley was conducted using two-pair primers: (1) forward primer (sdwF10) 5’ CTAGCTCACACACCTCTCATCTCAT 3’, and reverse primer (sdwR10) 5’ GTTCCCGACAAAAATTCCGTGT 3’; (2) forward primer (sdwF9) 5’ CTCTCCCGCACACTCACTCGCAAC 3’, and reverse primer (sdwR13) 5’ GCGGTGAGGGGGCATGCATAT 3’. PCR reaction was comprised of 50 ng of template DNA, 0.3 μM of each primer, 1 × PCR buffer, 0.4 mm dNTP and 0.2 U PCR enzyme (KOD FX, TOYOBO) in a final volume of 10 μl. PCR cycling conditions consisted of an initial denaturation step of 94 °C for 3 min, followed by 30-35 cycles of 98 °C for 10 s, 60 °C for 30s, and 68 °C for 3 min.

Primers of Morex_contig_40861 and neighbor genes of *HvGA20ox*_*2*_ were listed in Additional file 1: Table S1. PCR reaction was comprised of 1 × Taq Mix (Bio life), 0.3 μM of each primer and 50 ng of template DNA. The following PCR amplification profile was used : denaturation at 94 °C for 3 min, followed by 35 cycles of 94 °C for 30 s, 55 °C for 30 s, 72 °C for 0.5-2 min depending on the size of amplilcons, and a final extension at 72 °C for 5 min. The amplification products were run in 1 % agrose gels and sequenced by Biosune Biotechnology Co. Ltd.

### Plasmid construction and *Arabidopsis* transformation

For plasmid construction, full-length CDS of *HvGA20ox*_*2*_ was amplified from Bomi by PCR. The primers were designed as 5’ AGTACTCGAGCTCACACACCTCTCATCTCAT 3’ and 5’CTATGGATCCGAATCAGCCCGTGGAT 3’ with XhoI and BamH I sites, respectively. The amplified PCR product was confirmed by sequencing and cloned into T4 vector for further manipulation. Insert was confirmed by sequencing. The CDS paragraph of *HvGA20ox*_*2*_ digested with XhoI/ BamH I was cloned into the XhoI/ BamH Isite located between the 35S CaMV promoter and ocs terminator of the pFGC5941 binary vector, named as pFGC5941- *HvGA20ox*_*2*_. The binary vector was transformed into *Agrobacterium tumefaciens* by electroporation, and the floral dip method was used to transform Col-gl1 (Columbia ecotype, different from Col-o only in its glabrous leaf. Hereafter refer to wild type) and a semi-dwarf mutant *ga5-3* (a T-DNA inserted mutant of *AtGA20ox*_*1*_ in Col-o, Salk016701) [34]. T1 transgenic plants were screened by spraying bialaphos solution twice at an interval of three days at cotyledon stage, and confirmed by PCR using following specific primers: Forward, 5’ GGAGCATCGTGGAAAAAGAAGA 3’ (from CaMV 35S promoter sequence) and Reverse, 5’ GGAGTCGCAGGGCTGGTGTCC 3 ’(from *HvGA20ox*_*2*_). The constructs were also verified by sequencing. For analysis of *HvGA20ox*_*2*_ gene expression level in transgenic plants, 7-day-old Col-gl1, *ga5-3* and T3 transgenic seedlings were harvested for RNA extraction.

### Growth conditions and phenotypic characterization of *Arabidopsis*

The seeds of *Arabidopsis* were stratified at 4 °C for 1-2 d and sown on soil at 24 °C under LDs (16 h of light). Plants for hypocotyl and root length measurements were grown vertically on one half times Murashige and Skoog media (Sigma, M6899) containing 1 % MES, 3 % sucrose and 0.46 % Gelrite with pH 5.8 under LDs, being measured after 7 d. Flowering time was scored as the days when buds could be detected with naked eyes. Other measurements were performed on the plants that had stopped flowering.

### Real-time quantitative RT-PCR

RNA was extracted from the stems of Bomi and Riso no. 9265, as well as 7-day-old seedlings of T3 transgenic lines, Col-gl1 and *ga5-3* using Spin Column Plant total RNA Purification Kit(Sanggon Biotech (Shanghai) Co., Ltd). cDNA was prepared from 1 μg RNA using AMV First Strand cDNA Synthesis Kit(Sanggon Biotech (Shanghai) Co., Ltd). qPCR reactions were performed using SYBR Green (SG Fast qPCR Master Mix(High Rox), BBI ) and the Applied Biosystems Stepone plus Real-time PCR System. The Real-time PCR assays were performed in triplicate for each cDNA sample. For determining transcription levels of barley *GA20ox*_*2*_ and genes encoding final biosynthesis of GA, *HvACTIN* and *HvGAPDH* for barley, and *At1g13320* and *At4g26410* for *Arabidopsis* were employed as reference genes [5, 35, 36]. Additional file 2: Table S2 listed the oligonuleotide sequences used for quantitative RT-PCR.

### ELISA assay of gibberellin (GA_1+3_ and GA_4+7_)

Approximately 0.5 g fresh *Arabidopsis* tissue (7-day-old seedling) was homogenized in liquid nitrogen and extracted at 4 °C in cold 80 % (v/v) methanol containing 1 mM butylated hydroxytoluene for 4 h. The extracts were collected after centrifugation at 8000 g at 4 °C for 20 min. The residues were suspended in the same extraction solution and stored at 4 °C for 1 h, and then centrifuged again at 8000 g at 4 °C for 20 min. The two resulting supernatants were combined and passed through a C_18_ Sep-Pak cartridge (Waters, Milford, MA, USA). The efflux was collected and dried in N_2_. The residues was then dissolved in 0.01 M phosphate buffer solution (pH 7.5) and concentrations of GA_1+3_ and GA_4+7_ were determined in an indrect enzyme-linked immuno sorbent assay (ELISA) using GA_3_ and GA_4_ antibody, respectively. The ELISA KIT was obtained from Professor Baomin Wang (Chinese Agricultural University) and the methods were described in previous publications [37, 38].

### Statistical analyses

Phenotypic differences of Bomi and Riso no. 9265 were tested by Student’s *t* test. In order to avoid heterogeneity of variance, a natural log transformation was applied to hypocotyl length,and a square transformation was applied to flowering time (days), plant height, root and internode length. Least significant difference (LSD) at 5 % probability was used to assess the difference between genotypes. For statistical analysis of qPCR data, cycle threshold (C_T_) values were used to determine *Δ* C_T_ values (*Δ* C_T_ = C_T__target_ –C_T__reference_), and expression levels of target genes relative to reference gene were determined as 2^-*Δ* CT^. For comparison of GA concentration and the expression levels of GA-regulated transcripts between transgenic and wild type plants, ANOVA was used. Analysis of qRT-PCR efficiency showed that all amplicons of the genes used in this study were within the optimal range of 98.7-102.4 %.

### Ethics Statement

In this study we only used plant material including barley and Arabidopsis. Based on the rule of BMC Genomics, no ethics statement was required for the collection of genetic material.

## Additional files

Additional file 1:
**Table S1.** Primers used to detect flanking genes or sequences of *HvGA20ox*_*2*_. (XLS 18 kb) Additional file 2:
**Table S2.** Oligonucleotide sequences used in qRT-PCR assays. (XLS 17 kb)
